# The synergistic mechanism of multimodal psychological intervention in neurological rehabilitation and motor function recovery: from evidence-based practice to digital transformation

**DOI:** 10.3389/fpsyg.2025.1599133

**Published:** 2025-12-17

**Authors:** Xiang Xu, Zhengjing Fu

**Affiliations:** Changzhou Dean Hospital, Changzhou, Jiangsu, China

**Keywords:** multimodal psychological intervention, neurological rehabilitation, motor function recovery, evidence-based practice, digital transformation

## Abstract

With the development of the field of neurological rehabilitation, psychological intervention has gradually gained attention as an important adjunctive therapeutic approach in the recovery of motor function. Multimodal psychological intervention integrates psychology, exercise science, and digital health technologies, effectively promoting patients’ rehabilitation processes. Current research indicates that psychological intervention can not only improve patients’ emotional states but also enhance their sense of self-efficacy, thereby facilitating the recovery of motor function. However, despite some achievements in evidence-based practice, there remain numerous challenges in practical application, such as insufficient personalized interventions tailored to the needs of different patients and the complexity of integrating digital technologies. This review aims to explore the synergistic mechanisms of multimodal psychological intervention in depth, analyze its specific applications in neurological rehabilitation and motor function recovery, elucidate its fundamental theoretical framework, and anticipate the potential impact of digital transformation on the future development of this field, with the aim of providing effective guidance for clinical practice.

## 1 Introduction

Neurological rehabilitation and motor function recovery are important research areas in modern medicine, particularly in the treatment of conditions such as stroke and traumatic brain injury, where comprehensive rehabilitation programs are crucial for improving patients’ quality of life. In recent years, with the ongoing exploration of neurological rehabilitation methods, multimodal psychological intervention has emerged as an innovative therapeutic approach that has attracted widespread attention. However, it is important to situate this approach within the broader international and historical context of rehabilitation science. Multimodal psychological interventions, while increasingly recognized in recent years, are built upon decades of established practice and research on multidisciplinary rehabilitation teams in Western countries. For instance, the American Congress of Rehabilitation Medicine (ACRM) has long advocated for integrated approaches involving neuropsychologists, physical, occupational, and speech therapists in cognitive rehabilitation, establishing evidence-based recommendations for clinical practice (Cicerone et al., 2000). Similarly, the Commission on Accreditation of Rehabilitation Facilities (CARF) has standardized interdisciplinary care as a benchmark for quality rehabilitation since the 1960s, requiring the involvement of professionals from medical, psychological, and social domains (Commission on Accreditation of Rehabilitation Facilities, 1966). This review aims to bridge the current exploration of multimodal psychological interventions with this rich international legacy, analyzing its specific applications in neurological rehabilitation and motor function recovery within a globally informed theoretical framework.

The core of multimodal psychological intervention lies in its integrative nature, involving knowledge from multiple fields such as psychology, exercise science, and neuroscience (Jiang et al., 2025), and is aligned with the long-standing multidisciplinary team (MDT) model prevalent in international rehabilitation practice. Research shows that psychological factors play a significant role in neurological rehabilitation (Mascanzoni et al., 2024). The mental health status directly affects patients’ rehabilitation processes and outcomes. For instance, studies have found that improvements in psychosocial functioning are closely related to the recovery of motor function, with increased engagement and adaptability serving as important mediators for motor function enhancement (Mann et al., 2023). This finding emphasizes that psychological intervention should be an indispensable part of the neurological rehabilitation process, in addition to physical therapy, a principle long embedded in Western standards of care.

Moreover, multimodal psychological intervention has also shown positive effects in promoting neural plasticity (Guan et al., 2024). By strengthening patients’ psychological adaptability and emotional management, it can effectively promote the reorganization and functional recovery of the nervous system. For example, research indicates that combining exercise training with psychological intervention can significantly enhance motor function and quality of life in stroke patients (McLoughlin, 2023). This combination not only improves patients’ sense of self-efficacy but also enhances their awareness of active participation in the rehabilitation process, thus promoting better rehabilitation outcomes.

With the development of digital technologies, the implementation of multimodal psychological intervention is gradually transitioning to digital transformation. By utilizing emerging technologies such as virtual reality (VR) and augmented reality (AR), patients can engage in psychological and exercise training in more realistic and interactive environments. This novel intervention approach not only increases patient engagement but also allows for real-time monitoring and feedback on the rehabilitation process, further optimizing treatment plans (Kandhari et al., 2022). For instance, the application of brain-computer interface (BCI) technology provides patients with new avenues for rehabilitation by enabling them to interact directly with computer systems, thereby improving their control over movement and enhancing the recovery of neural function (Pichiorri and Mattia, 2020).

In summary, multimodal psychological intervention demonstrates significant synergistic mechanisms in neurological rehabilitation and motor function recovery. By comprehensively considering patients’ physiological, psychological, and social factors, this intervention approach not only helps improve patients’ motor abilities but also enhances their overall quality of life. In the future, as digital technologies continue to develop, multimodal psychological intervention is expected to play a larger role in clinical practice, bringing new transformations to the field of neurological rehabilitation.

## 2 Basic theoretical framework of multimodal psychological intervention

The concept of multidisciplinary team (MDT) approaches in rehabilitation has a long history, dating back to Hellenic antiquity and evolving through the 20th century with contributions from figures like John Bonica in pain management and Cicely Saunders in palliative care (Karassava et al., 2024). In modern practice, MDTs are considered the gold standard in rehabilitation, integrating medical, psychological, and social dimensions to address complex patient needs. The field of Rehabilitation Psychology has further systematized the integration of psychological principles into neurological rehabilitation, emphasizing the role of psychologists in promoting coping skills, functional outcomes, and quality of life (Cicerone et al., 2000; Commission on Accreditation of Rehabilitation Facilities, 1966). Multimodal psychological intervention, as discussed in this review, is a contemporary embodiment of this established multidisciplinary philosophy, tailored for neurological populations.

### 2.1 Definition and classification of psychological intervention

Psychological intervention refers to various psychological methods and techniques aimed at improving individuals’ mental health and behavioral performance (D’Onofrio et al., 2022). Based on different theoretical foundations and application contexts, psychological interventions can be classified into various types, including cognitive—behavioral therapy, psychodynamic therapy, family therapy, group therapy, and psychoeducation. In recent years, with an increasing emphasis on mental health, the forms of psychological intervention have become increasingly diverse, especially in the field of neurological rehabilitation, where more studies indicate that multimodal psychological interventions can effectively enhance patients’ rehabilitation outcomes (Schupp, 2011).

Multimodal psychological intervention typically combines multiple therapeutic approaches, such as psychological counseling, exercise therapy, nutritional intervention, and social support, to achieve the comprehensive improvement of patients’ psychological and physiological states (Hall et al., 2024).

### 2.2 The relationship between neural plasticity and psychological factors

Neural plasticity refers to the ability of the nervous system to reorganize and adapt after experiencing external stimuli or internal changes (Zheng et al., 2014). This process plays an important role in mental health, particularly in individuals who have experienced trauma or chronic stress. Research shows that psychological factors such as emotional states, stress levels, and social support can all influence the expression of neural plasticity (Tatarinova et al., 2020). For example, patients with depression often exhibit reduced neural plasticity, while positive psychological interventions can promote neural plasticity, improving patients’ emotional and cognitive functions (Fiorindi et al., 2024). Furthermore, structured cognitive rehabilitation, as recommended by evidence-based guidelines from bodies like the American Congress of Rehabilitation Medicine, has been shown to promote neural plasticity through targeted cognitive and behavioral strategies (Cicerone et al., 2000). Psychological intervention can promote neural remodeling in the brain by improving individuals’ emotional states and coping mechanisms, helping patients better adapt to challenges and stress in their lives (Demurtas et al., 2024).

### 2.3 Implementation strategies for multimodal psychological intervention

The implementation strategies for multimodal psychological intervention typically include the following aspects: first, developing individualized intervention plans based on patients’ specific needs and psychological states to choose appropriate intervention methods. Second, combining various therapeutic approaches, such as cognitive-behavioral therapy, exercise therapy, and nutritional intervention, to form a comprehensive treatment plan aimed at enhancing the effectiveness of the intervention and patient engagement (Amano et al., 2023). Additionally, regularly assessing patients’ psychological states and rehabilitation progress to timely adjust intervention strategies to ensure the sustainability and effectiveness of treatment (Huang et al., 2021). Research shows that patients who undergo multimodal psychological intervention exhibit significant improvements in mental health and quality of life, indicating a broad application prospect for this strategy in the field of neurological rehabilitation (Hall et al., 2024).

### 2.4 Applications of multimodal psychological intervention in neurological rehabilitation

#### 2.4.1 The impact of psychological support on rehabilitation willingness

Psychological support plays a crucial role in the neurological rehabilitation process, particularly regarding patients’ willingness to engage in rehabilitation. Research indicates that psychological support can significantly enhance patients’ willingness to participate in rehabilitation plans, thereby promoting their overall recovery (Choudhury et al., 2006). For example, a study focusing on patients with chronic pain found that those receiving psychological intervention showed significant improvements in rehabilitation willingness and quality of life (Rivano Fischer et al., 2021). Additionally, psychological support can help patients overcome fears and anxieties associated with the rehabilitation process, boosting their confidence in the outcomes of rehabilitation. By establishing a good doctor-patient relationship, the medical team can provide necessary emotional support to patients, thereby increasing their engagement and adherence.

In neurological rehabilitation, patients often face the loss of physical function and psychological stress, leading to feelings of helplessness and frustration during the rehabilitation process. The intervention of psychological support can not only alleviate these negative emotions but also enhance patients’ willingness to rehabilitate by increasing their sense of self-efficacy. Research shows that after receiving psychological support, patients become more actively involved in rehabilitation activities, which in turn improves rehabilitation outcomes (Padmavathi et al., 2023). Therefore, medical teams should recognize the role of psychological support and design corresponding psychological intervention programs to promote patients’ willingness to rehabilitate and their engagement.

#### 2.4.2 The role of social interaction in patient rehabilitation

Social interaction is also of significant importance in patients’ rehabilitation processes. Studies show that a strong social support network can significantly improve rehabilitation outcomes for patients, particularly in the field of neurological rehabilitation (Bove et al., 2020). Social interaction not only provides emotional support but also enhances patients’ sense of participation and belonging through group activities, thereby promoting their rehabilitation. For example, research involving patients with mental illnesses found that good social relationships were closely related to patients’ rehabilitation progress, with social interaction effectively alleviating anxiety and depressive symptoms (Linde et al., 2023).

In neurological rehabilitation, patients often need to confront the recovery of physical functions and the adjustment of psychological states, and social interaction can provide them with necessary support. By participating in group activities, patients can receive encouragement and support from others while learning from the rehabilitation experiences of peers, thus enhancing their confidence and motivation for rehabilitation (Deng et al., 2024). Furthermore, social interaction can improve patients’ quality of life, helping them adapt better to life after rehabilitation (Kouidi, 2004). Therefore, when the medical team develops rehabilitation plans, they should consider how to promote social interaction among patients to enhance their rehabilitation outcomes.

#### 2.4.3 Combined strategies of exercise and psychological interventions

The combination of exercise and psychological interventions has shown good effects in neurological rehabilitation. Research indicates that integrating physical exercise with psychological interventions can significantly improve patients’ rehabilitation outcomes, particularly in enhancing mental health and physical function (Estrella-Castillo and Gomez-de-Regil, 2016; Fan et al., 2024). For instance, a study on patients with heart disease showed that exercise training combined with psychological intervention effectively reduced patients’ anxiety and depression levels while improving their cardiopulmonary function and quality of life (Zheng and Zhao, 2024).

The effectiveness of this combined strategy lies in the fact that exercise not only improves physiological functions but also enhances patients’ psychological states by releasing neurochemicals such as endorphins.

During exercise, patients can effectively relieve stress and anxiety while experiencing the pleasure that physical activity brings, enhancing their sense of self-efficacy. Furthermore, psychological interventions can help patients overcome fear and resistance to exercise, making them more willing to participate in physical activities, thus achieving dual improvements in both physical and psychological aspects (Sripada and Walters, 2023).

Therefore, when designing rehabilitation programs, the medical team should integrate exercise with psychological interventions to create personalized comprehensive intervention plans that maximize patients’ rehabilitation effects. This multimodal approach not only promotes the recovery of physical functions but also improves patients’ mental health and enhances their overall quality of life.

### 2.5 Key factors in exercise function recovery

#### 2.5.1 Exercise training and neural adaptation mechanisms

Exercise training plays a crucial role in neurological rehabilitation and recovery of motor functions (Tanaka et al., 2020).

Research has shown that exercise training can not only improve physical strength and endurance but also activate neural adaptation mechanisms, thereby enhancing neural plasticity. Neural adaptation refers to the changes in the nervous system’s responsiveness to exercise stimuli, involving neuronal reorganization and synaptic strengthening. Exercise training can enhance neural adaptability by increasing nerve conduction velocity, improving connections between neurons, and promoting the release of nerve growth factors. For example, after systematic exercise training, patients’ motor abilities and coordination significantly improved, which is closely related to the activation of neural adaptation mechanisms (Hall et al., 2024).

Moreover, exercise training can promote neuronal survival and functional recovery by improving blood flow and oxygen supply. Studies show that regular exercise can increase cerebral blood flow and promote the secretion of neurotrophic factors (such as BDNF), which play a key role in neuronal growth and survival (Amano et al., 2023). Thus, exercise training is not only a means of physical rehabilitation but also an essential strategy for promoting the recovery of neural functions.

#### 2.5.2 The promoting role of cognitive function in exercise recovery

Cognitive function also plays an important role in exercise recovery. Research indicates that improvements in cognitive abilities can directly affect exercise performance and rehabilitation outcomes. Cognitive functions include attention, memory, and executive functions; enhancements in these abilities can help patients better understand and execute exercise training instructions, thereby improving training effectiveness (Angiolillo et al., 2023). For instance, in one study, participants who underwent cognitive training showed significant improvements in their motor abilities and daily living activities, indicating that improvements in cognitive function can promote exercise recovery (Fiorindi et al., 2024).

Additionally, cognitive improvement is closely related to mental health. A good psychological state can enhance patients’ motivation and sense of participation, thus improving the effectiveness of exercise training. Research has found that patients with good mental health exhibit higher persistence and positivity during exercise recovery, directly promoting their motor function recovery (Demurtas et al., 2024). Therefore, considering the combination of cognitive functions and exercise training will help formulate more effective rehabilitation plans.

#### 2.5.3 The impact of mental health on rehabilitation outcomes

Mental health plays an indispensable role in the recovery of exercise functions. Research indicates that psychological health issues such as anxiety and depression significantly affect patients’ rehabilitation outcomes, leading to decreased motor abilities and prolonged recovery times (Fiorindi et al., 2024). For example, patients with depression often exhibit low motivation and high fatigue during exercise rehabilitation, making it difficult for them to persist in training, which in turn affects rehabilitation outcomes (Perna and Harik, 2020).

On the other hand, a good mental health state can enhance patients’ self-efficacy and motivation for rehabilitation, thus promoting the recovery of motor functions. Research has found that patients who participate in psychological interventions and supportive therapies show significantly better recovery of motor functions compared to those who do not receive psychological interventions (Hall et al., 2024). Therefore, when formulating rehabilitation plans, considering patients’ mental health status and providing corresponding psychological support will help improve rehabilitation outcomes.

In summary, exercise training, cognitive function, and mental health are key factors affecting the recovery of motor functions. By comprehensively considering these factors, more effective individualized rehabilitation strategies can be developed to enhance patients’ motor functions and quality of life.

### 2.6 Research findings in evidence-based practice

#### 2.6.1 Recent advances in clinical trials

In recent years, the application of multimodal psychological interventions in neurological rehabilitation and recovery of motor functions has gradually attracted researchers’ attention. The latest clinical trials indicate that these interventions can significantly improve patients’ functional recovery and quality of life (Jiao et al., 2024; Dominguez-Tellez et al., 2020). For instance, a study on patients with concussions found that an intervention plan combining exercise training, cognitive behavioral therapy, and psychological support effectively shortened patients’ recovery time and enhanced their daily living capabilities (McLoughlin, 2023). Additionally, research has shown that patients undergoing multimodal interventions exhibit significant improvements in motor capacity, cognitive function, and psychological state, particularly in the recovery of motor functions, showing higher clinical effectiveness (Kakudate et al., 2021).

Meanwhile, an increasing number of clinical trials have adopted randomized controlled designs to ensure the reliability and effectiveness of the results. For example, in a study targeting stroke patients, researchers compared the effects of traditional single interventions with multimodal interventions and found that the latter had significant advantages in improving motor functions and mental health (De Leo et al., 2021). These studies not only provide evidence-based support for clinical practice but also lay the foundation for future intervention strategies.

#### 2.6.2 Empirical data supporting intervention effects

The accumulation of empirical data provides solid support for the effectiveness of multimodal psychological interventions. Multiple studies have systematically assessed intervention effects, finding that multimodal interventions can significantly enhance patients’ motor abilities and mental health levels. A study targeting patients with depression showed that the intervention group combining exercise and psychological therapy significantly outperformed the control group receiving only single treatment in reducing depressive symptoms (Toomey and Jalbert, 2021). Furthermore, another study indicated that patients undergoing multimodal interventions progressed significantly faster in functional recovery compared to traditional treatment methods (Campbell et al., 2024).

Further analysis revealed that the enhancement of intervention effects was closely related to patients’ engagement and the personalized design of interventions. Research has found that regular feedback mechanisms can effectively improve patients’ sense of involvement and adherence, thereby promoting rehabilitation outcomes (Roberto de Barros et al., 2023). For instance, in a study involving patients with dementia, researchers adjusted intervention plans through feedback mechanisms, resulting in significant improvements in both cognitive function and daily living abilities (Sandberg, 2024).

#### 2.6.3 Continuous monitoring and feedback mechanisms

Continuous monitoring and feedback mechanisms play a crucial role in multimodal psychological interventions. By real-time monitoring of patients’ rehabilitation progress, clinicians can timely adjust intervention strategies to meet individual patient needs (Pritwani et al., 2024). For example, in a study targeting patients with chronic pain, researchers used smart devices to monitor patients’ pain levels and psychological states in real time, adjusting treatment plans based on feedback information, which significantly improved patient satisfaction and treatment outcomes (Seguchi et al., 2025).

Moreover, feedback mechanisms can enhance patients’ self-management capabilities. Research shows that participants receiving continuous feedback in interventions can better understand their rehabilitation progress, thereby enhancing their confidence and adherence to treatment (Kim et al., 2024). For example, in a study involving diabetes patients, researchers provided real-time health monitoring and feedback through a mobile application, helping patients better manage their condition and ultimately achieve significant health improvements (Shang et al., 2024).

In conclusion, continuous monitoring and feedback mechanisms not only enhance the effectiveness of interventions but also promote patients’ active participation, providing a successful implementation of multimodal psychological interventions. Strong assurance: With the continuous advancement of technology, future intervention programs will focus more on personalization and real-time feedback in order to achieve better rehabilitation outcomes.

### 2.7 The impact of digital transformation on psychological interventions

#### 2.7.1 Integration of digital health tools

With the rapid development of digital technology, the application of digital health tools in psychological interventions is becoming increasingly widespread. These tools include mobile applications, wearable devices, and online platforms, which can effectively support patients’ self-management and mental health. Research shows that digital health tools can not only increase patient engagement in treatment but also enhance their sense of self-efficacy, thereby improving mental health status (Nahm et al., 2025). For example, one study found that patients using digital health tools demonstrated higher motivation for self-monitoring and behavior change, resulting in better health outcomes (McLoughlin, 2023). In addition, the personalized features of digital health tools allow interventions to better adapt to the specific needs of patients, thus improving the effectiveness of interventions (Kraepelien et al., 2023).

The integrated application of digital health tools is also reflected in their combination with traditional treatment methods. By integrating digital tools with face-to-face psychotherapy, patients can receive ongoing support and feedback during the treatment process. This combination not only enhances the flexibility of treatment but also helps patients continue self-management after the treatment ends, thereby reducing the risk of relapse (Gentile et al., 2022).

However, despite the significant potential of digital health tools, their implementation still faces many challenges, including technology acceptance, privacy protection, and data security issues (Tan et al., 2024).

#### 2.7.2 Remote interventions and patient self-management

The application of remote interventions in the field of mental health has gradually become a trend, especially during the COVID-19 pandemic, when many psychological interventions shifted to remote modes. Research shows that remote interventions can effectively enhance patients’ self-management abilities, particularly regarding mental health issues (Castellano-Tejedor and Cencerrado, 2024). Through video consultations, telephone therapy, and online support groups, patients can receive professional psychological support in a comfortable environment, thus reducing the psychological barriers associated with face-to-face consultations (Park et al., 2025).

The advantages of remote interventions lie in their accessibility and flexibility, allowing more patients to obtain the psychological support they need. For example, remote interventions for patients with depression have shown effects similar to traditional face-to-face treatments, and patients have a high level of acceptance for this format (Hasel et al., 2024). Furthermore, remote interventions can help patients better manage their mental health through real-time data monitoring and feedback, enhancing their sense of self-efficacy and self-regulation abilities (Wathne et al., 2025).

However, remote interventions also face challenges, including technological barriers, patients’ digital literacy, and the emotional support that may be lacking due to the absence of face-to-face interaction (Leal Neto and Von Wyl, 2024). Therefore, when designing remote interventions, it is essential to fully consider these factors to ensure the effectiveness of the intervention and patient satisfaction.

#### 2.7.3 The role of data analysis in personalized interventions

Data analysis plays a crucial role in personalized psychological interventions. By conducting in-depth analysis of patients’ health data, healthcare providers can identify specific needs and behavioral patterns of patients, allowing for the development of more precise intervention plans (Castellano-Tejedor and Cencerrado, 2024). For instance, using machine learning and artificial intelligence technologies, researchers can analyze large volumes of health data to identify key factors affecting patients’ mental health and adjust intervention strategies accordingly (Barclay et al., 2025).

The core of personalized interventions lies in the ability to dynamically adjust treatment plans based on patient feedback and data. This flexibility not only enhances the effectiveness of the intervention but also increases patients’ sense of involvement and satisfaction (Doumen et al., 2023). For example, one study showed that by monitoring patients’ mental states and behavioral changes in real-time, therapists could promptly adjust treatment plans to improve treatment outcomes (Bober et al., 2024).

Additionally, data analysis can help evaluate the effectiveness of interventions, providing a basis for future research. By comparing the effects of different intervention strategies, researchers can identify the most effective intervention methods, thereby promoting further development in the field of mental health (Kilfoy et al., 2025). However, issues of data privacy and security remain significant considerations in the implementation of personalized interventions and must be addressed during the design and implementation process (Longhini et al., 2025).

## 3 Conclusion

In today’s rapidly evolving healthcare environment, the application of multimodal psychological interventions in the fields of neural rehabilitation and motor function recovery has gradually become a research hotspot. This intervention method not only combines the theoretical foundations of psychology but also integrates the practical experience of sports science, providing patients with a more comprehensive and systematic rehabilitation plan. By analyzing existing research findings, it is clear to see the significant synergistic effects of multimodal interventions in enhancing rehabilitation outcomes.

Firstly, the impact of psychological factors on physical recovery has been widely recognized. Numerous studies have shown that a positive psychological state can promote patients’ physical recovery, while negative emotions may lead to a decline in rehabilitation outcomes. Therefore, incorporating psychological interventions into the rehabilitation process can effectively improve patients’ emotional states, thereby promoting the recovery of motor functions. This finding provides important theoretical support for clinical practice, encouraging more rehabilitation centers to adopt a model that integrates psychological interventions to enhance overall rehabilitation outcomes.

Secondly, digital transformation offers new opportunities for the implementation of multimodal psychological interventions. The use of digital tools and platforms, such as virtual reality and mobile health applications, can achieve personalized intervention plans, enhancing patient engagement and initiative. These technologies can not only help patients manage their rehabilitation process better but also monitor patients’ recovery status in real-time through data analysis, providing scientific evidence for clinicians. Future research should continue to explore how to better utilize these digital technologies to optimize intervention effects.

However, despite the promising outlook of multimodal psychological interventions, attention must be paid to the differences in viewpoints and findings among different studies. Some studies may focus on a specific psychological intervention method or be conducted within particular patient populations, leading to limitations in results. Therefore, future research should strengthen comparative analyses of different intervention methods and explore their applicability and effectiveness in diverse populations. Additionally, interdisciplinary collaboration will be key to advancing this field, as the joint efforts of psychologists, sports scientists, and clinicians will help build a more comprehensive and scientific rehabilitation system.

In summary, the importance of multimodal psychological interventions in neural rehabilitation and motor function recovery is increasingly evident. They not only bring new hope to patients but also provide innovative directions for medical practice. With further in-depth research, we look forward to forming more systematic intervention measures to meet the needs of different patients and achieve higher-quality rehabilitation outcomes. In this process, emphasizing the balance and integration of different research perspectives will be an important assurance for the continued development of this field.
